# Cleavage of Hemagglutinin-Bearing Lentiviral Pseudotypes and Their Use in the Study of Influenza Virus Persistence

**DOI:** 10.1371/journal.pone.0106192

**Published:** 2014-08-28

**Authors:** Olivier Sawoo, Amélie Dublineau, Christophe Batéjat, Paul Zhou, Jean-Claude Manuguerra, India Leclercq

**Affiliations:** 1 Institut Pasteur, Environment and Infectious Risks Research and Expertise Unit, Laboratory for Urgent Response to Biological Threats, Paris, France; 2 University of Paris Diderot, Sorbonne Paris Cité (Cellule Pasteur), Paris, France; 3 Institut Pasteur of Shanghai, Unit of Antiviral Immunity and Genetic Therapy, Chinese Academy of Sciences, Shanghai, China; University of Georgia, United States of America

## Abstract

Influenza A viruses (IAVs) are a major cause of infectious respiratory human diseases and their transmission is dependent upon the environment. However, the role of environmental factors on IAV survival outside the host still raises many questions. In this study, we used lentiviral pseudotypes to study the influence of the hemagglutinin protein in IAV survival. High-titered and cleaved influenza-based lentiviral pseudoparticles, through the use of a combination of two proteases (HAT and TMPRSS2) were produced. Pseudoparticles bearing hemagglutinin proteins derived from different H1N1, H3N2 and H5N1 IAV strains were subjected to various environmental parameters over time and tested for viability through single-cycle infectivity assays. We showed that pseudotypes with different HAs have different persistence profiles in water as previously shown with IAVs. Our results also showed that pseudotypes derived from H1N1 pandemic virus survived longer than those derived from seasonal H1N1 virus from 1999, at high temperature and salinity, as previously shown with their viral counterparts. Similarly, increasing temperature and salinity had a negative effect on the survival of the H3N2 and H5N1 pseudotypes. These results showed that pseudotypes with the same lentiviral core, but which differ in their surface glycoproteins, survived differently outside the host, suggesting a role for the HA in virus stability.

## Introduction

Influenza A viruses (IAVs) cause a serious worldwide public health problem that can lead to severe illnesses and deaths through yearly epidemics [1] and pandemics [2]. Similarly, pandemic threats with new IAV strains such as the H1N1(2009) pandemic virus (H1N1pdm) [3], have stimulated numerous studies on the transmission mechanism of these viruses [4] [5] [6] [7]. However, the knowledge on how environmental factors may impact IAV persistence or their transmission is still rudimentary [8] [9]. Understanding these factors is critical for efficient decision-making during the emergence of new IAVs. We have previously shown that IAVs can persist in water and on surfaces for an extended period of time and that the susceptibility of the virus to a given temperature or salinity was not due to genomic degradation [10] [11]. Our findings suggested that external structures of the virions could play a role in viral persistence in the environment. Indeed, IAV is an enveloped virus which acquires its lipid bilayer with two embedded glycoproteins, the hemagglutinin (HA) and the neuraminidase (NA), by budding from the host cell membrane [12]. To complete the replication cycle of the virus, the homotrimeric HA undergoes a cleavage activation at a proteolytic or cleavage site by host cell proteases, a crucial step to yield fully infectious particles [13]. Cleavage of the HA precursor results into two subunits HA1 and HA2, exposing the hydrophobic fusion peptide at the N-terminus of HA2 which mediates entry of IAV into host cells by fusion of the viral bilayer with the cell endosomal membrane [14]. This cleavage is essential for virus infectivity and is important for influenza virus pathogenicity in avian hosts [13] [15]. Most influenza strains possess a monobasic cleavage site (MCS) which is cleaved by tissue-restricted proteases only, such as exogenous protease trypsin-clara or cell-associated proteases like type II transmembrane serine proteases (TTSPs) TMPRSS2, TMPRSS4 and human airway trypsin-like protease (HAT) [13]. Highly pathogenic H5 or H7 subtypes, on the other hand, contain a polybasic cleavage site (PCS) which is cleaved by the ubiquitous endogenous protease furin through the Golgi pathway [16]. Therefore, the entry in target cells requires a cleaved hemagglutinin protein in IAVs or any HA bearing system.

**Figure 1:**
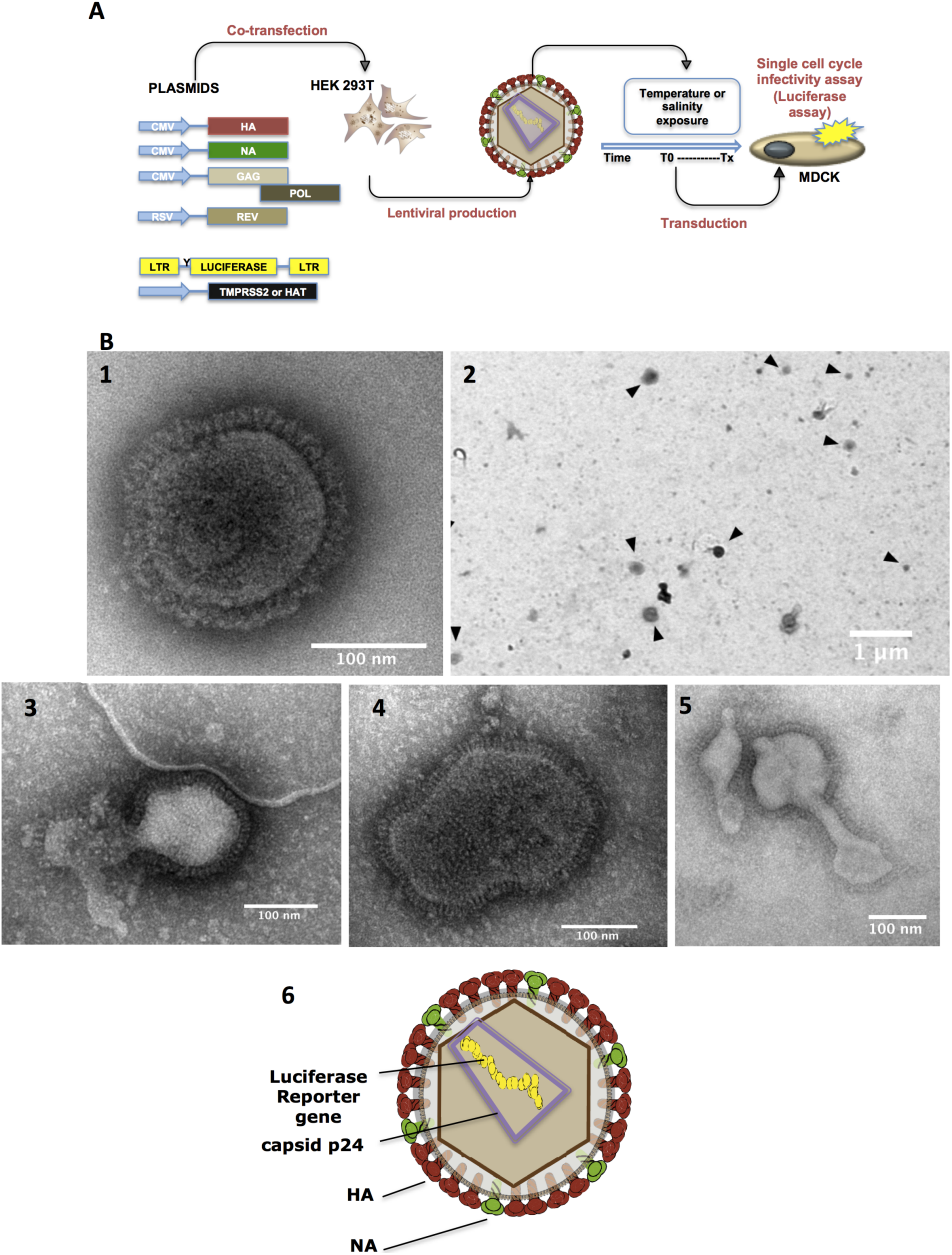
Production of lentiviral pseudotypes bearing hemagglutinin glycoproteins of influenza A virus and their use in survival kinetics. **A.** Schematic representation of lentiviral production. 293T cells were co-transfected with plasmids expressing HA and NA, plasmids coding for the lentiviral core (GAG-POL-REV), the encapsidated luciferase genome and HAT/TMPRSS2 proteases. For persistence studies, lentiviral pseudotypes were exposed to water at different temperatures or salinities. At different time intervals, infectivity was assessed through transduction of MDCK cells and luciferase assays. **B.** Transmission electronic micrographs. Spherical shape of an H1N1 influenza virus (1). Overview of lentiviral pseudotypes with black arrows representing spherical pseudoparticles (2). Negative stained of damaged (3), spherical (4) and pleiomorphic (5) pseudoparticles. Schematic representation of a HA/NA bearing lentiviral pseudotype (6).

In this work, we evaluated the use of IAV lentiviral pseudotypes as an experimental tool to study the impact of environmental factors on influenza virus survival as external structures such as the HA can easily be targeted through single-cycle infectivity assay (Figure 1A). The IAV pseudotype consists in a lentiviral core containing a reporter replication deficient genome, and bearing NAs and cleavage-dependent HAs on their surface (Figure 1B). Their use provides a safe tool to study highly pathogenic avian influenza (HPAI) glycoproteins in biosafety level 2 conditions. Lentiviral vectors are widely used but most of the previous works published with avian and human influenza virus pseudotypes were related to serological assays, drug discovery, vaccine study or diagnosis [17]. In this study, we investigated how different HAs, isolated from different IAV strains, may influence influenza virus survival through the use of lentiviral highly transduceable and cleavable pseudotypes. We showed that increasing temperature and salinity had a negative effect on the survival of the pseudotyped IAVs, as shown with their viral counterparts [10]. Moreover, differences in survival behaviour were observed for pseudotypes bearing HAs isolated from H1N1, H3N2 and HPAI H5N1 strains, suggesting that the nature of the hemagglutinin protein plays a role in the stability of IAV in the environment.

## Materials and Methods

### Cells and viruses

Madin Darby canine kidney (MDCK) cells were maintained in Minimum Essential Medium (MEM 1X, GIBCO, Life Technologies), supplemented with 10% foetal calf serum (FCS), tricine (10 mM, Sigma) and antibiotics (100 units.mL^−1^ penicillin, 100 µg.mL^−1^ streptomycin, GIBCO, Life Technologies). Human embryonic kidney 293T cells (ATCC CRL-3216) were maintained in Dulbecco's modified Eagle's medium with GlutaMAX (DMEM, GIBCO, Life Technologies) supplemented with 10% FCS and antibiotics (100 units.mL^−1^ penicillin, 100 µg.mL^−1^ streptomycin, GIBCO, Life Technologies). All cells were incubated at 37°C in humidified 5% CO_2_ incubator. For viral stock production, two H3N2, two H1N1 and two H5N1 strains of influenza A viruses: A/Victoria/3/75 (H3N2 VIC/75), A/Wisconsin/67/2005 (H3N2 WIS/05), A/New Caledonia/20/99 (H1N1 NC/99), pandemic A/Paris/2590/2009 (H1N1 PAR/09), A/Hong Kong/156/97 (H5N1 HK/97) and A/duck/Cambodia/D4(KC)/2006 (H5N1 CAM/06) respectively, were grown on MDCK cells without FCS and in the presence of 2 µg.mL^−1^ of trypsin-TPCK (Trypsin/L-1-Tosylamide-2-phenylethyl chloromethyl ketone, Whortington Biochemical Corporation) at 35°C for 3 days. The clarified supernatants were harvested, aliquoted and stored at –80°C.

### Plasmids

The lentiviral gag-pol and rev plasmids (pLP1 and pLP2 respectively) were isolated from the Virapower lentiviral expression kit (Invitrogen, Life Technologies) which contains a mixture of pLP1, pLP2, and pLP/VSVG plasmids, supplied in solution at 1 µg.µL^−1^ in TE Buffer, pH 8.0. XL1 blue supercompetent cells (Agilent technologies) were transformed with the plasmid mixture and pLP1 and pLP2 plasmids were selected and amplified after plating and sequencing of chosen bacterial colonies. The firefly luciferase expressing plasmid (pTRIP-LUC), which contains the HIV encapsidation signal, provided the encapsidated reporter genome [18]. pCMV-HA or -NA expression plasmids encoding the HA or the NA of A/Thailand/1(KAN–1)/2004 H5N1 human strain (H5 THAI/04) were used to subclone the HA and NA genes from the different viruses mentioned above [19] [20]. Briefly, viral RNA was extracted (Nucleospin Dx virus kit, Macherey Nagel) and reverse-transcribed (SuperScript III Reverse Transcriptase, Invitrogen, Life technologies) with UNI3 primer (5′-AGCAAAAGCAGG-3′). The HA and NA open reading frames were then amplified by PCR (Phusion Taq, Fermentas) with primers containing SalI and EagI restriction sites (primers are listed in Table 1). PCR products corresponding to the HA and NA genes were subcloned into the H5 THAI/04 pCMV-HA or pCMV-NA expression plasmids respectively.

**Table 1:** Sequences of the primers used in RT-PCR for cloning the HA and NA of the different IAV strains.

	HA	NA
UNI3	AGCAAAAGCAGG	
**H3N2** **VIC/75**	**F:** GTCGACACGATCCGATATCGCCGCCACCATGAAGACTATC	**F:** GTCGACACGATCCGATATCGCCGCCACCATGAATCCAAATCAAAAG
	**R:** TAGAGCGGCCGCTCAAATGCAAATGTTGCACC	**R:** TAGAGCGGCCGCTTATATAGGCATGAGATTGATGTCCGCCCC
**H3N2** **WIS/05**	**F:** GTCGACACGATCCGATATCGCCGCCACCATGAAGACTATC	**F:** GTCGACACGATCCGATATCGCCGCCACCATGAATCCAAATCAAAAG
	**R:** TAGAGCGGCCGCTCAAATGCAAATGTTGCACC	**R:** TAGAGCGGCCGCTTATATAGGCATGAGATTGATGTCCGCCCC
**H1N1** **NC/99**	**F:** GTCGACACGATCCGATATCGCCGCCACCATGAAAGCAAAACTACTGG	**F:** GTCGACACGATCCGATATCGCCGCCACCATGAATCCAAATCAAAAAATAATAACC
	**R:** TAGAGCGGCCGCTCAGATGCATATTCTACACTGC	**R:** TAGAGCGGCCGCCTACTTGTCAATGGTGAACGGC
**H1N1** **PAR/09**	**F:** GTCGACACGATCCGATATCGCCGCCACCATGAAGGCAATACTAGTAG	**F:** GTCGACACGATCCGATATCGCCGCCACCATGAATCCAAACCAAAAG
	**R:** TAGAGCGGCCGCTTAAATACATATTCTACACTG	**R:** TAGAGCGGCCGCTTACTTGTCAATGGTAAATGGC
**H5N1 CAM/06**	**F:** ACCGTCGTCGACGCCACCATGGAGAAAACAGTGCTTCTTCTTG	**F:** ACCGTCGTCGACGCCACCATGAATCCAAAT
	**R:** TAGAGCGGCCGCTTAAATGCAAATTCTGCATTGTAACGATCC	**R:** TAGAGCGGCCGCCTACTTGTCAATGGTGAATGG
**H5N1** **HK/97**	**F:** ACCGTCGTCGACGCCACCATGGAGAAAATAG	**F:** ACCGTCGTCGACGCCACCATGAATCCAAAT
	**R:** TAGAGCGGCCGCTTAAATGCAAATTCTGCA	**R:** TAGAGCGGCCGCCTACTTGTCAATGGTGAATGG

F: forward primers and R: reverse primers (5′-3′).

Sequences of the constructed wild-type or mutated pCMV-HA or NA plasmids were confirmed by sequencing.

To generate polybasic cleavage site (PCS) mutants, PCS listed in Table 2 were inserted into corresponding pCMV-HA plasmids by using Quickchange II mutagenesis kit (Agilent technologies). Primer sequences were designed using CLC BIO workbench software and are available upon request.

**Table 2:** Pseudotypes bearing HAs with different polybasic cleavage sites (PCS).

	Pseudotypes	Cleavage sites
**Wild Type**	H3N2 WIS/05	**NVPSI—QS———RG**
**PCS 1**	H3N2 WIS/05 PCS 1	**NVPSI—QS——RRRKKRG**
**PCS 2**	H3N2 WIS/05 PCS 2	**NVP——Q—RERRRKKRG**
**PCS 3**	H3N2 WIS/05 PCS 3	**NVPEK Q——RRRKKRG**
**PCS 4**	H3N2 WIS/05 PCS 4	**NVP——Q——RRRKKRG**
**PCS 4**	H3N2 VIC/75 PCS 4	**NVP——Q——RRRKKRG**
**PCS 4**	H1N1 PAR/09 PCS 4	**NVP——Q——RRRKKRG**
**Wild Type**	H5N1 THAI/04	**NVP——Q—RERRRKKRG**

### Generation of lentiviral vectors

Lentiviral pseudotypes bearing influenza HA and NA (Figure 1A) were generated by co-transfecting 6×10^5^ 293T cells with 2 µg of pCMV-HA, 0.5 µg of NA, 3.5 µg of pLP1, 3.5 µg of pLP2, and 3.5 µg of pTRIP-LUC using fugene HD (Promega) according to the manufacturer’s instructions. Forty-eight hours post-transfection, the cell supernatants were clarified, harvested, filtered through 0.45 µm Polytetrafluorethylene (PTFE) Membrane Filter (Sartorius AG) and stored at –80°C.

### Cleavage of HA-bearing pseudotypes

For cleavage of HA-bearing pseudotypes by exogenous proteases, various concentrations of Trypsin-TPCK were applied for 30 min before MDCK cells transduction followed by the addition of Soybean trypsin inhibitor according to the manufacturer’s instructions (Sigma-Aldrich).

For endogenous cleavage by TMPRSS2 or HAT proteases, 2 µg of pNeo3-TMPRSS2 or 1 µg of pNeo3-HAT (generous gifts from Dr Mikhail Matrosovich, Institute of Virology, Philipps University, Germany) were co-transfected during pseudotype production.

### Chloroquine and sodium butyrate treatment

Briefly, 2 to 4 hours before transfection, 293T cells were supplemented with 25 µM of chloroquine (Chloroquine diphosphate salt, Sigma-Aldrich), followed by the addition of 10 µM sodium butyrate (Sigma-Aldrich) 8 hours post-transfection. Twenty-four hours after treatment with sodium butyrate, the medium was substituted with fresh medium and pseudotypes were harvested 24 hours later.

### Hemagglutination and hemolysis assay

Hemagglutination assay was performed using 50 µL of influenza pseudotypes and 50 µL of a 0.5% suspension of guinea pig red blood cells (Charles Rivers) as described previously [21]. The HA titer was expressed in “hemagglutination unit” (HAU).

For hemolysis assay, 100 µL of pseudotypes or viruses diluted (1∶2) in 100 µL of phosphate buffered saline (PBS, Life technologies) were mixed with 200 µL of a 2% suspension of guinea pig red blood cells and incubated at 37°C for 10 min. After clarification, the pellets were re-suspended in PBS at pH = 5, and further incubated at 37°C for 10 min. The absorbance at 540 nm was thereafter measured.

### Antibodies

Sheep HA-antisera (anti-H3N2 WIS/05, anti-H3N2 VIC/75 or anti-H1N1 PAR/09, from NIBSC), rabbit anti-TMPRSS2 (LifeSpan BioSciences) or mouse anti-p24 (Abcam) were used as primary antibodies (1∶1000 dilution). Anti-sheep conjugated with either horseradish peroxidase or FITC (1∶10000 or 1∶2000 dilutions respectively, Jackson ImmunoResearch), anti-mouse conjugated with Allophycocyanin (1∶2000, Jackson ImmunoResearch) or anti-rabbit conjugated with FluoProbes 547 (1∶2000 dilution, InterChim) were used as secondary antibodies.

### Western blotting

One hundred microliters of pseudotypes or influenza virus were denatured in Laemli’s buffer (4X, Biorad) with 10% 2-mercaptoethanol (Biorad) at 95°C for 5 min. Fifty microliters of protein extracts were separated by SDS-PAGE (4–15% polyacrylamide TGX gel, Biorad) and subsequently transferred to a PVDF membrane (Biorad). The expression of the different proteins was detected by incubation with 1∶1000 diluted primary antibodies (HA or p24 specific) and 1∶10000 diluted species-specific peroxidase-conjugated secondary antibodies. Proteins were visualised with ECL plus peroxidase substrate (Pierce) following the manufacturer’s instructions and exposure to auto-radiographic films during 1 min (GE healthcare). Protein p24 was used as loading control.

### Transmission Electron Microscopy (TEM)

Two milliliters of pseudotypes or influenza viruses were concentrated on 1000 MWCO Vivaspin column (Sartorius) or were ultracentrifuged for 2 hours at 25000 g on a 20% sucrose cushion using a TLS-55 (Optima TLX Ultracentrifuge, Beckman). Ten microliters were put on a microscopy copper grid (Sigma Aldrich) and fixed with 2% (v/v) paraformaldehyde (PFA, Electronic microscopy sciences) for 5 min. Samples were then negatively stained with 5 µL of phosphotungstic acid (Sigma Aldrich) and observed under a transmission electron microscope (CM-10, Philips).

### Single-cycle infectivity assay

For single-cycle infectivity assays with pseudotypes, 3.3×10^5^ MDCK cells in 96-well plate were transduced with 100 µL of harvested pseudotypes. Three days later, adherent cells were washed in PBS and submitted to 100 µL of Glo lysis Buffer (Promega) at room temperature for 1 hour. Twenty microliters of cell lysates were assayed for luminescence using the Luciferase assay system (Promega) (as described in the manufacturer’s manual) on a Tristar2 Berthold luminometer. Titers were expressed in relative luminescence units (RLUs).

### Persistence study using lentiviral pseudotypes

Pseudotypes produced in the absence of trypsin, chloroquine or sodium butyrate but with TMPRSS2 and HAT proteins, were removed from −80°C, diluted (1∶2) in distilled water (Braun) and exposed to different temperatures (4°C, 25°C and 35°C) or salinities (0 and 35 g.L^−1^ of NaCl, Fluka, Sigma). At different time points, single-cycle infectivity assays were performed through transduction of MDCK cells with 100 µL of inoculated water samples (Figure 1A). Cells were assayed for luminescence 72 hours after MDCK cells transduction (as described above).

### Immunofluorescence microscopy

Transfected 293T cells on a glass coverslip were washed three times with PBS, fixed for 10 min with 4% paraformaldehyde and permeabilised with 0.25% Triton X-100 (Sigma) in PBS containing 10% FCS. Cells were stained for 1 hour with 1∶1000 dilution of primary antibody, followed by incubation with the respective secondary antibodies (see above). All washing steps were performed with 500 µL of PBS three times. Coverslips were then mounted with Prolong Gold anti-fade containing DAPI (Invitrogen) and observed under an inverted Apotome fluorescence microscope (Observer Z1, ZEISS).

### Quantitative Real Time RT-PCR (qRT-PCR)

Luciferase encapsidated RNA was extracted using Nucleospin Dx Virus kit (Macherey-Nagel) from 150 µL of pseudotype suspension, according to the manufacturer's instructions. Quantitative RT-PCR targeting the luciferase gene was carried out using the following primers [forward 5′-ACACCCCAACATCTTCGAC-3′, Reverse 5′-TCGCGGTTGTTACTTGACTG-3′] and probe [5′-FAM-TTGGAGCACGGAAAGACGATGAC-BHQ1-3′] (BHQ1: black hole quencher 1) with a LightCycler 480 instrument (Roche) and a SuperScript III Platinum OneStep RT-PCR kit (Invitrogen). The reactions were incubated in a 96-well optical plate at 45°C for 15 min, then 95°C for 3 min, followed by 50 cycles of 95°C for 10 sec, 50°C for 10 sec and 72°C for 20 sec and a cooling step at 40°C for 30 sec.

### P24 Capsid ELISA

Lentiviral pseudotype titer was determined by p24 ELISA following the manufacturer’s instructions (Clontech).

### Statistical analyses

Individual variables were compared between groups with a one-way or two-way ANOVA as appropriate. Student’s test (unpaired) was used to test differences between specific means. Significance was set at p<0.01. All experiments were carried out in triplicate, values were expressed as means unless specified otherwise and error bars indicated standard deviations.

## Results

### Production of lentiviral HA-bearing pseudotypes

Lentiviral pseudotypes were produced by co-transfecting 293T cells with the relevant plasmids as described in material and methods (Figure 1A). Around 80% of the pseudotypes were spherical and 110–250 nm in diameter with hemagglutinin spikes of 9–11 nm in size on their surface (Figures 1B), which is similar to influenza A virions (Figure 1B (1)). In addition, few pleomorphic pseudotypes were also observed as well as some damaged particles (Figures 1B). In order to optimize pseudotype titers, we tested the addition of sodium butyrate and chloroquine on lentiviral pseudotype production as it was previously described for other retroviral vectors [22]. Efficiency of H5-pseudotype production in the presence of chloroquine, sodium butyrate or both, was either quantified by qRT-PCR targeting the encapsidated luciferase gene, or by single-cycle infectivity assays through transduction of MDCK cells (Figure S1). All experiments were carried out in triplicate and were repeated independently twice. Co-transfecting the different plasmids in the presence of sodium butyrate or chloroquine increased the lentiviral production titer, but did not exceed a 10-fold yield increase for the genomic titer and less than a 5-fold increase for the log_10_ RLU titer. In single-cycle infectivity assays, the use of negative controls HA-/NA- and PBS yielded a 3 log_10_ RLU signal which corresponded to the background limit of the technique. A slight increase in lentiviral production was observed after action of both treatments simultaneously. This increase in lentiviral production was confirmed by an enhancement in the hemagglutination capacity of the pseudotypes (Table S1).

### Exogenous cleavage of H3 and H1 pseudotypes with trypsin-TPCK

**Figure 2:**
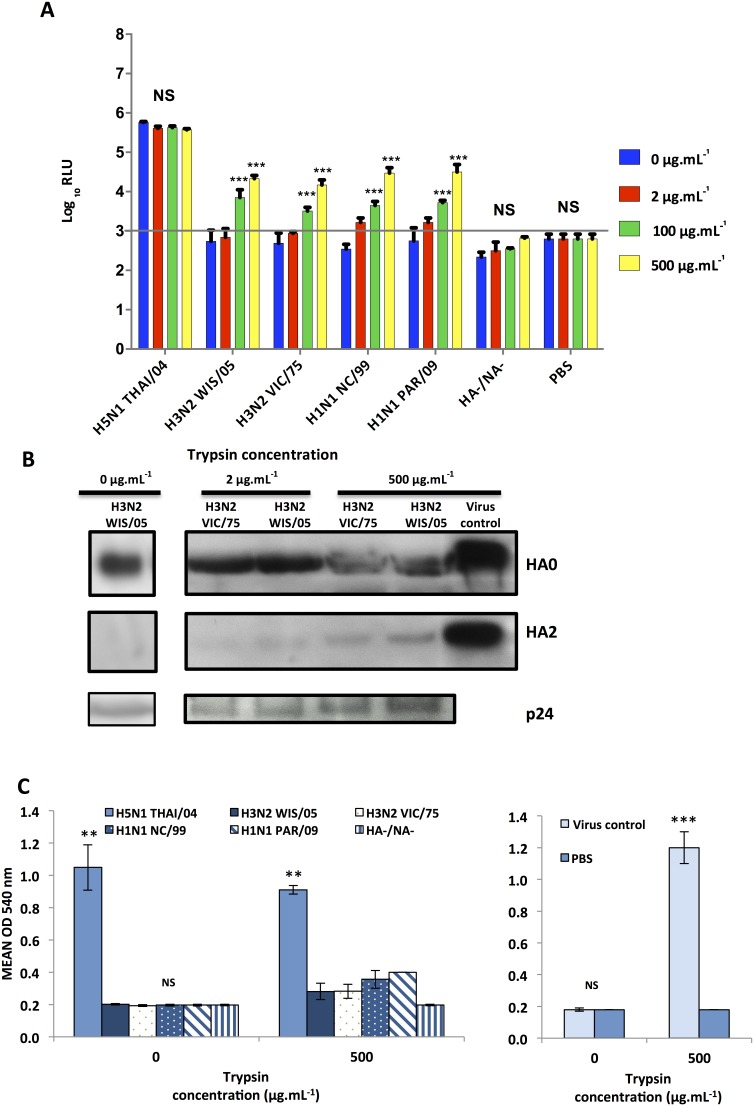
Cleavage of H3 and H1 pseudotypes with trypsin-TPCK. **A.** Pseudotypes were exposed to different concentrations of trypsin (0, 2, 100 and 500 µg.mL^−1^) for 30 min followed by single-cycle infectivity assays and luciferase assays (expressed in Mean log_10_ Relative Luminescence Units, RLU) with a minimum value of 3.0 log_10_ RLU, which corresponded to the background limit (grey line). Data are expressed as the mean ± Standard Deviation (S.D) of triplicate repeats. ***p<0.0001 versus 0 µg.mL^−1^ concentration, NS (not significant), Student's t-test. **B.** SDS-PAGE/Western blot analysis of pseudotypes bearing different HAs after treatment with trypsin-TPCK. The H3N2 WIS/05 virus was used as a positive control (virus control). Western blot was performed using antibodies targeting the HA protein (HA0 and HA2 representing uncleaved and cleaved HA respectively) or the HIV p24 (loading control). **C.** Hemolysis assays were performed on different pseudotypes after treatment with 0 or 500 µg.mL^−1^ trypsin-TPCK. Lysis of guniea pig red blood cells was evaluated by measuring the optical density of released haemoglobin at 540 nm. The H3N2 WIS/05 virus was used as a positive control (virus control) and PBS (phosphate buffered saline) as a negative control. Data are expressed as the mean ± S.D of triplicate repeats. ***p<0.0001, **p<0.001 and NS (not significant), Student's t-test.

One major problem with influenza-based pseudotypes is the cleavage of the MCS bearing hemagglutinin. The cleavage of H1 or H3 hemagglutinins by the exogenous protease trypsin-TPCK was assessed by exposing pseudotypes to different concentrations of trypsin, followed by single-cycle infectivity assays. H5 pseudotypes were used as controls being independent of trypsin cleavage, and pseudotypes without HA and NA (HA-/NA-) and PBS were used as negative controls. H1 and H3 pseudotypes were cleaved in a dose-dependent manner, and higher proteolytic cleavage was observed with extreme concentration of trypsin as high as 100 or 500 µg.mL^−1^ (Figure 2A). However, even with high concentration of trypsin, more than 1.5 log_10_ RLU fold change was observed when H5 lentiviral production was compared to H1 or H3 pseudotypes. This suggested that a substantial amount of H3 or H1 hemagglutinin was not cleaved. Indeed, western blot analysis showed that a large amount of HA0 (uncleaved HA) was still present when compared to HA2 fragments (Figure 2B). Hemolysis assays gave similar results (Figure 2C) even with high concentration of trypsin in comparison to cleaved virus. These results showed the inefficiency of trypsin-TPCK to fully cleave H1 and H3 hemagglutinins from lentiviral pseudotypes even with high concentration of trypsin. To improve proteolytic cleavage, PCS sequences were inserted in H3 or H1 hemagglutinins to mimic H5 pseudotypes.

### Polybasic cleavage site insertion in H3 and H1 pseudotypes

**Figure 3:**
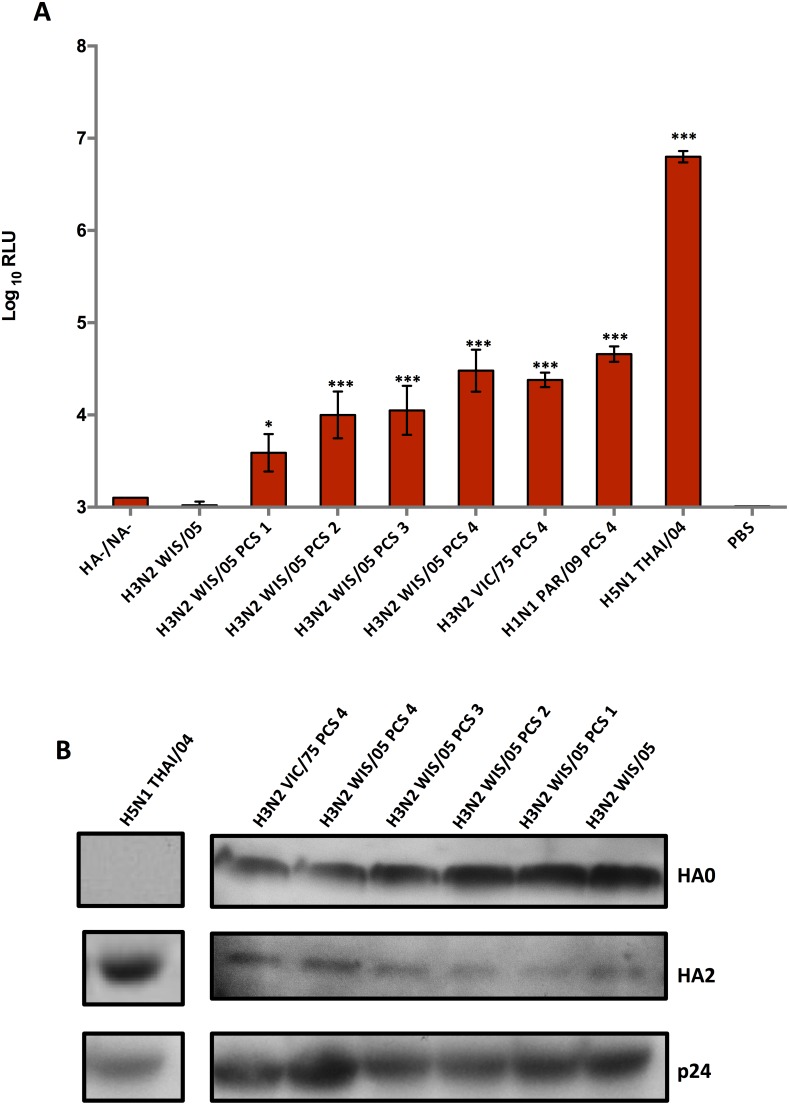
Single-cycle infectivity assays with H3 and H1 pseudotypes containing a polybasic cleavage site (PCS). **A.** Single-cycle infectivity assays were performed with different PCS pseudotype mutants in the absence of trypsin-TPCK. The H3N2 WIS/05 (virus control) or HA/NA- pseudotypes and PBS were used as negative controls. Data are expressed in log_10_ RLU (background limit; 3 log_10_ RLU) as the mean ± S.D of triplicate repeats. ***p<0.0001, *p<0.01 and NS (not significant), Student's t-test. **B.** Western blot analysis of the PCS pseudotype mutants in the absence of trypsin-TPCK was performed using anti-HA serum specific to the H3 serotype (HA0 and HA2 representing uncleaved and cleaved HA respectively). H5N1 THAI/04 pseudotypes were used as a positive control.

We evaluated the influence of the presence of a PCS on the cleavability of H3 or H1 hemagglutinnin bearing pseudotypes. Four PCS, which differed in length or neighbouring amino acids were inserted into either H3 or H1 HAs expression plasmids by site-directed mutagenesis (Table 2). Cleavage of the corresponding mutant pseudotypes was assessed after transduction of MDCK cells by luciferase assays (Figure 3A). H3 and H1 PCS pseudotype mutants showed significantly more cleavage than wild-type H3N2 pseudotypes (H3N2 WIS/05). Unexpectedly, all pseudotyped mutants produced in the absence of trypsin-TPCK showed more than a 100-fold reduction of infectivity (RLU) compared to H5 pseudotypes. RLU profiles obtained with the different PCS mutants were similar to those produced in the presence of trypsin-TPCK (data not shown). In accordance to the RLU assays, western blot analysis showed a high proportion of uncleaved PCS HA0s in opposition to H5N1 THAI/04 pseudotypes, which were totally cleaved (Figure 3B). Thus, the simple addition of a PCS in a hemagglutinin sequence did not result in optimal efficient cleavage by endogenous furin in the absence of trypsin.

### Cleavage of H3 and H1 pseudotypes by endogenous co-transfected proteases

**Figure 4:**
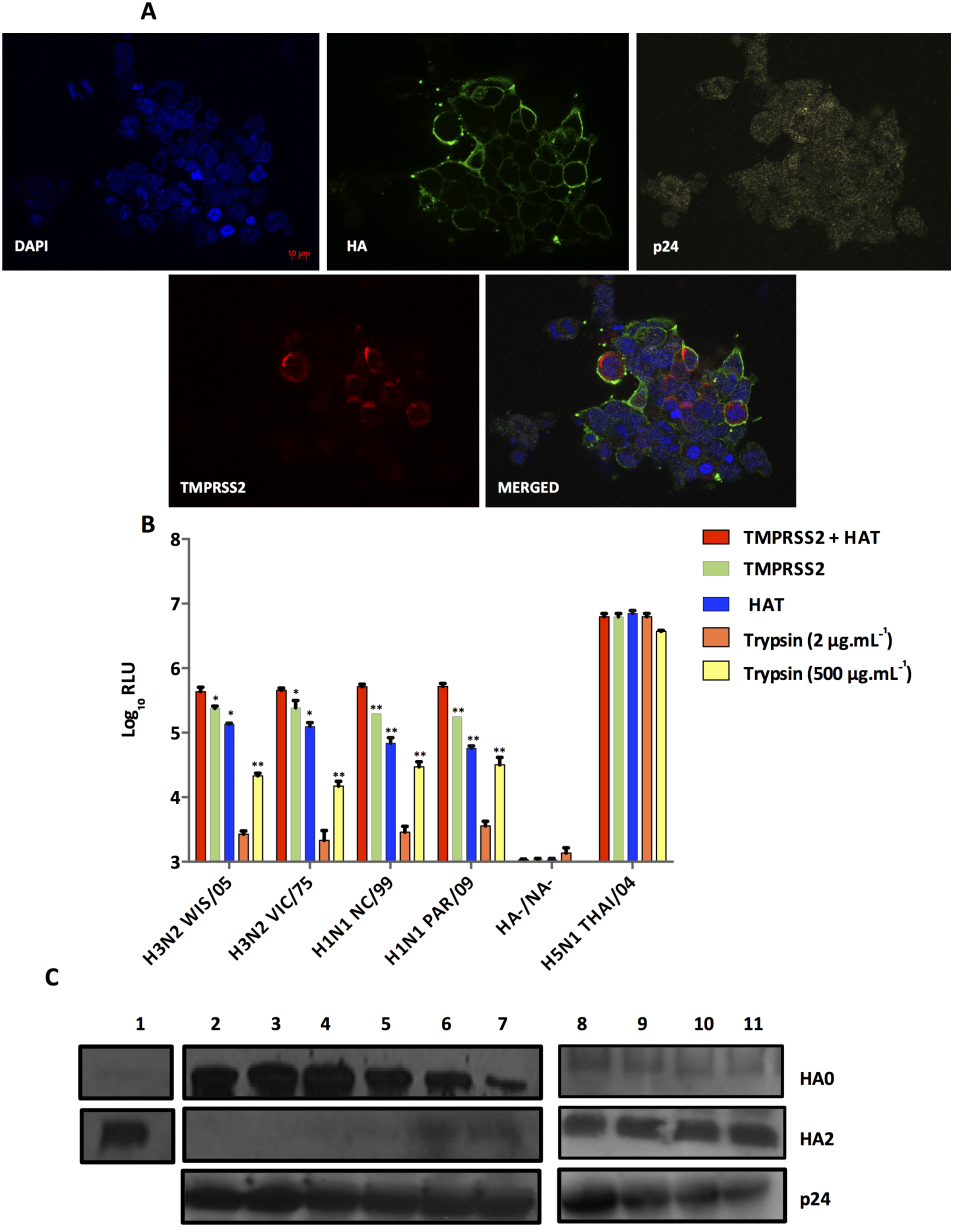
Cleavage of H3 and H1 pseudotypes with TMPRSS2 and HAT proteases. **A.** Subcellular localization of HA, p24 and TMPRSS2 proteins in transfected HEK 293T cells by structured-illumination fluorescent microscopy. **B.** Pseudotypes were produced in the presence of trypsin-TPCK or HAT or TMPRSS2, or TMPRSS2 and HAT followed by single-cycle infectivity assays (expressed as the mean log_10_ RLU ± S.D of triplicate experiments (background limit; 3 log_10_ RLU). *p<0.01 **p<0.001, and ***p<0.0001 (unpaired Student’s t-test). **C.** SDS-PAGE and western blot analysis were performed on pseudotypes produced in the presence of TMPRSS2 and HAT, using a mixture of sera specific to H3 and H1 serotypes with HA0 and HA2 representing uncleaved and cleaved HA respectively (Lane 8: H3N2 WIS/05, lane 9: H3N2 VIC/75, lane 10: H1N1 PAR/09, lane 11: H1N1 NC/99). The H3N2 WIS/05 virus was used as a positive control (Lane 1). Uncleaved H3N2 WIS/05 and H1N1 PAR/09 pseudotypes (Lanes 2 and 3 respectively) were used as negative controls. H3N2 WIS/05 and H1N1 PAR/09 pseudotypes were cleaved with either 2 µg.mL^−1^ of trypsin-TPCK (Lanes 4 and 5 respectively) or with the HAT protease alone (Lanes 6 and 7 respectively).

Recent studies have shown that the use of trypsin-like proteases such as HAT or TMPRSS2 could improve the cleavage of HAs [23]. We thus transfected plasmids coding for HAT or/and TMPRSS2 during lentiviral production (Figure 1A). Expression of HA, TMPRSS2 and p24 was monitored by structured-illumination immunofluorescence microscopy (Figure 4A). HA and p24 proteins were detected in 100% of cells and TMPRSS2 protein in approximately less than 50% of the cells tested. HA and TMPRSS2 staining were exclusively localised near the lipid bilayer, while p24 showed cytoplasmic and peri-cytoplasmic membrane sub-localisations. The cleavage of H1 or H3 pseudotypes was evaluated through single-cycle infectivity assays. As shown in figure 4B, the presence of serine proteases HAT or TMPRSS2 resulted in better cleavage of H1 or H3 pseudotypes than with trypsin-TPCK (2 or 500 µg.mL^−1^) with a difference as high as 50-fold and even up to 200-fold. When both serine proteases were used, we observed a 3-fold increase of mean log_10_ RLUs compared to the effect of HAT or TMPRSS2 alone while no significant effect was observed with H5N1 THAI/04 pseudotypes. The efficient cleavage of H1 or H3 pseudotypes was also confirmed by western blot analysis (Figure 4C). These results showed that the use of trypsin-independent proteases such as HAT and TMPRSS2 greatly increased the titer of cleaved H1 or H3 pseudotypes allowing their use in persistence studies. As a result, for persistence studies, H1 or H3 bearing pseudotypes were produced in the presence of both HAT and TMPRSS2 proteins.

### Persistence of influenza pseudotypes in water

**Figure 5:**
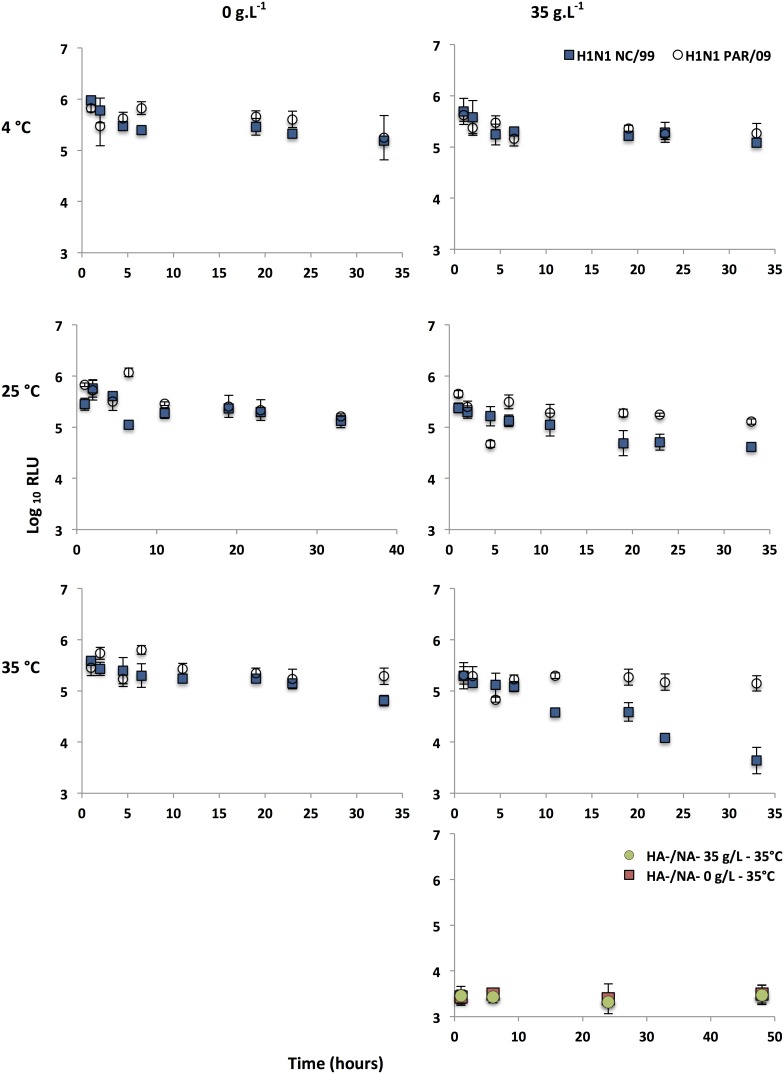
Persistence of H1 pseudotypes in water. H1N1 pseudotypes were diluted in water (1∶2) in the presence (35 g.L^−1^) or absence (0 g.L^−1^) of NaCl and were exposed to 4, 25, and 35°C. At each time interval (represented in hours), single-cycle infectivity assays were performed (represented in mean log_10_ RLU and background limit; 3 log_10_ RLU). All experiments were carried out in triplicate. Pseudotypes without HA and NA proteins on their surface (HA-/NA-) were used as a negative control. Error bars indicate standard errors; **p<0.001 and ***p<0.0001 (unpaired Student’s t-test).

**Figure 6:**
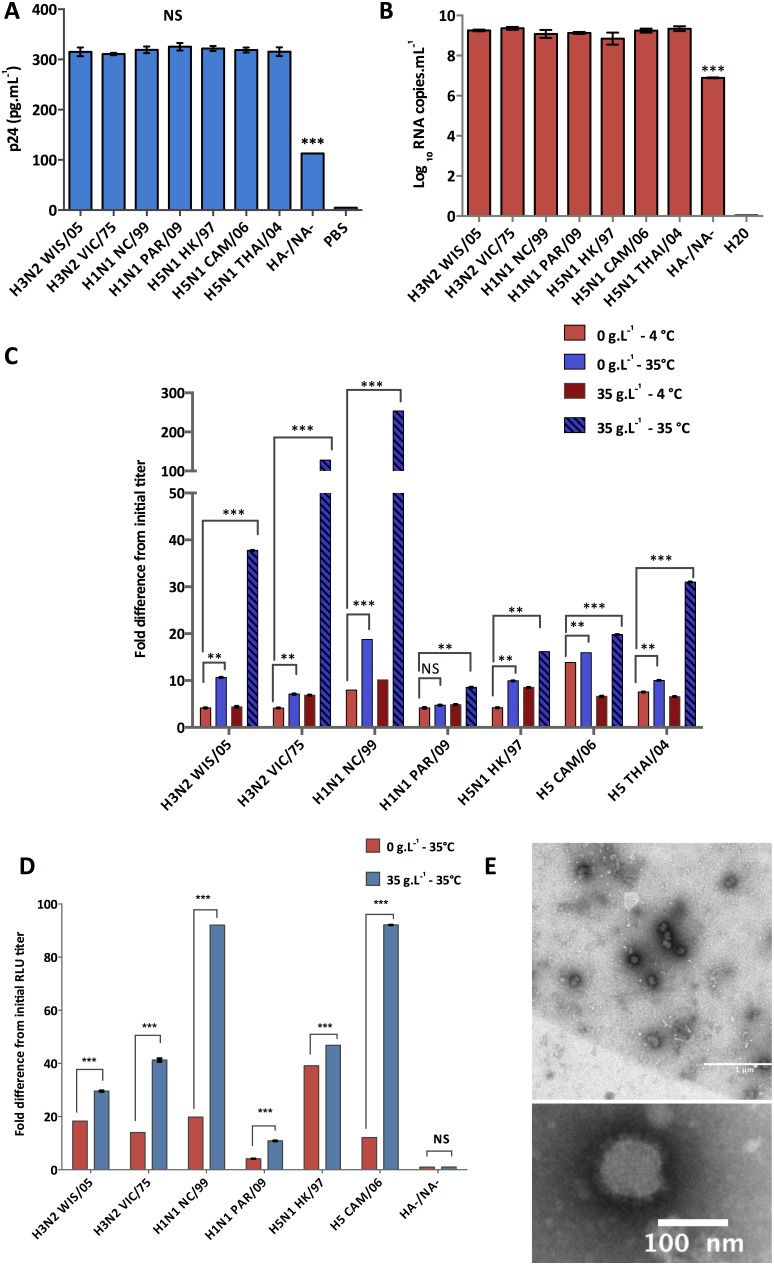
Persistence of H1, H3, and H5 pseudotypes in water. **A.** P24 capsid ELISA was performed on three independent production of HA bearing pseudotypes in triplicate. PBS and pseudotypes produced in the absence of HA and NA proteins were used as negative controls (PBS and HA-/NA- respectively). **B.** Quantitative real-time RT-PCR targeting the luciferase genome was performed on three independent production of HA bearing pseudotypes (expressed as the mean Log_10_ RNA copies.mL^−1^ ± SD of triplicate repeats). H_2_0 and pseudotypes produced in the absence of HA and NA proteins were used as negative controls (H_2_0 and HA-/NA- respectively). **C.** Pseudotypes were diluted in water (1∶2) in the presence (35 g.L^−1^) or absence (0 g.L^−1^) of NaCl and were exposed to 4 and 35°C. At each time interval, single-cycle infectivity assays were performed. Loss of infectivity was estimated by calculating the difference between the RLUs after 48 hours and the initial RLU titer. **D.** Pseudotypes from two independent production stocks were diluted in water (1∶2) in the presence (35 g.L^−1^) or absence (0 g.L^−1^) of NaCl and were exposed to 35°C. At each time interval, single-cycle infectivity assays were performed. Loss of infectivity was estimated by calculating the difference between the RLUs after 48 hours and the initial RLU titer. Experiments were carried in triplicates and error bars indicate standard errors of the mean; ***p<0.0001 and NS (not significant) (unpaired Student’s t-test). **E.** Transmission electronic microcopy of negatively stained H1 pseudotypes, which have been exposed to 35°C and 35 g.L^−1^ of NaCl for 72 hours.

To investigate the potential role of the HA in virus stability, pseudotypes bearing different HAs were subjected to water at 3 different temperatures (4, 25 or 35°C, representing cold, temperate and hot climates respectively) and different salinities (0 or 35 g.L^−1^, representing salinity of freshwaters or average salinity of oceans respectively). Single-cycle infectivity assays were performed at different time intervals (Figure 1A). Infectivity of seasonal H1N1 NC/99 and pandemic H1N1 PAR/09 pseudoparticles decreased over time, with only a slight log_10_ RLU variation for cold temperature such as 4°C (Figure 5). However, at 35°C and/or 35 g.L^−1^ of NaCl, seasonal H1N1 NC/99 pseudotypes were more affected than the H1N1pdm pseudotypes. Similar experiments were performed with pseudotypes bearing HAs from different subtypes (HPAI H5N1 or H3N2) (survival kinetics in Figure S2 and S3). We first evaluated the amount of pseudotypes generated during each production before using them in persistence studies. The lentiviral pseudoparticle titer was evaluated using p24 ELISA (Figure 6A) and qRT-PCR targeting the luciferase gene (Figure 6B). For each strain, no significant differences were observed between three independent pseudotype productions whereas a lower amount of p24 or reporter genome was detected for pseudotypes produced without HA and NA (HA-/NA-). Although budding is independent of the presence of envelope proteins during HIV infection [24], the lack of envelope proteins may result in intracellular trafficking disruption and thus cause a decrease in HA-/NA- pseudotypes production as shown previously with polarized cells [25]. The loss of infectivity of H3, H1 and H5 pseudotypes was then estimated by calculating the difference between the RLUs after 48 hours and the initial RLU titer at 4 and 35°C, in the absence or presence of salt (0 or 35 g.L^−1^ respectively) (Figure 6C). These results showed that high temperature and/or salinity had a strong negative effect on HA bearing pseudotype viability. Interestingly, at high temperature and salinity, differences were higher with H3N2 and seasonal H1N1 pseudotypes (up to 100-fold and 250-fold difference respectively). A similar negative effect was also observed with H5N1 pseudotypes but with lower differences (up to 30-fold difference). In order to assess if this difference was linked to the presence of a PCS, monobasic or polybasic bearing H3N2 pseudotypes were cleaved by serine proteases and then exposed to 35°C and 35 g.L^−1^ of NaCl. MCS or PCS H3N2 pseudotypes displayed similar survival trends, which reflected no difference due to the insertion of a PCS in the HA protein (Figure S4). In addition, H3, H5 and H1 pseudotypes from 2 independent productions were subjected to 35°C in water, with a concentration of salt of 0 or 35 g.L^−1^. The loss of infectivity was then evaluated (Figure 6D) by calculating the difference between the RLUs after 48 hours and the initial RLU titer. These results confirmed the negative impact of salinity and temperature on influenza pseudotypes with higher fold differences with H3N2 and H1N1 NC/99 pseudotypes. Because pH variation could be an important bias in loss of infectivity, the pH was also monitored for each aliquot used to estimate the RLU titer and was found to be stable (6.8 to 7.3). TEM analysis performed on pseudotypes which have been exposed to high temperatures and salinities showed undamaged enveloped pseudovirions (Figure 6E). Moreover, quantitative genomic evaluation studies were also carried out by performing qRT-PCR targeting the encapsidated luciferase genome, at each time point for all samples. Results showed that genome was not degraded overtime (data not shown). Our results are in agreement with those obtained with IAVs in similar conditions, where no loss of viral genome was observed while there was a decrease in IAV infectivity [10], [11].

Altogether, the results presented here showed the involvement of the nature of the HA protein in the loss of infectivity of lentiviral pseudotypes, suggesting a similar implication in the case of IAVs.

## Discussion

Influenza-based lentiviral vectors were used in a wide range of applications such as gene transfer [26], in diagnosis with neutralisation assays [27] [28], in high throughput screening for antiviral discovery [29] or in immunisation studies [30]. In contrast, this tool was less used for studying the molecular biology of influenza lifecycle. In this study, we used an influenza pseudotyped system to investigate the potential role of the HA in IAV survival outside the host. TEM analysis of the pseudovirions showed that their shape, their size and the density of their surface glycoproteins were similar to what has been previously observed with H5 pseudotypes [31]. Moreover, on a comparable basis, it was also relevant to use a model that does not differ from influenza virions neither in shape nor in size [32]. Although IAV survival could not be strictly compared to lentiviral pseudoparticle survival, the latter provides a simple tool where viral external structures of IAV were tested independently from internal influenza proteins. In this study, different HA bearing lentiviral pseudotypes were subjected to different temperatures or salinity.

For efficient production of pseudotypes and their use in survival kinetics, the initial titer of the pseudotypes is critical. Hence, optimization of the lentiviral production was needed as demonstrated by previous studies [17] [28]. In this study, we tested the use of chloroquine, a drug which is known to inhibit lysosomal degradation of transfected DNA [33] [34] and sodium butyrate, which enhances retroviral production [22]. Altogether, the results in this experiment showed that the combined use of sodium butyrate and chloroquine was associated with a slight improvement in vector production in both infectivity assays (RLU) and genomic titer. Given these results, this protocol was not applied for the production of pseudotypes used in persistence studies.

Proteolytic cleavage was also a crucial step when non-polybasic HA bearing pseudotypes were produced. Trypsin-TPCK is widely used to cleave the HA of IAVs during in vitro amplification of these viruses. Various concentrations were tested, ranging from 2 µg.mL^−1^ of exogenous trypsin-TPCK, which is a frequent concentration, to concentrations as extreme as 500 µg.mL^−1^. However, complete cleavage of pseudotypes could not be achieved even with the use of 500 µg.mL^−1^ of trypsin. This inefficiency of trypsin to cleave influenza pseudotypes was described before [35] but with concentrations tested not exceeding 50 µg.mL^−1^. One possible hypothesis for partial cleavage is the inaccessibility of surface HAs to trypsin or the trypsin inhibition by the FCS used as a supplement during lentiviral production. Indeed, in order to maintain active cells and obtain high titered pseudotypes, we used culture medium supplemented with 10% of FCS in contrast to MCS bearing IAVs which were produced in FCS-free medium with low concentration of trypsin. When FCS was removed or its concentration reduced, lower infectivity was observed after MDCK transduction (data not shown). 293FT cells, a clonal isolate derived from HEK 293T cells, which is less dependent on the presence of FCS was used for lentiviral production. Transduction experiment and western blot analysis revealed a 10% rise in cleavage in the presence of high concentration of trypsin-TPCK, but there was still a large proportion of uncleaved HA0s (data not shown). However, the total content of p24 protein detected was lower, which reflected a decrease in overall production of lentiviral pseudotypes without FCS. This was confirmed by the quantification of the luciferase gene (data not shown). Taken together, these results showed that removal of FCS and the use of high concentration of trypsin should lead to the production of cleaved H3 or H1 pseudotypes, but with low overall titers. Moreover, this would lead inevitably to use trypsin inhibitors followed by incubation at 37°C to avoid transduced cells detachment which could impact on the survival kinetics of the pseudotypes. To overcome this cleavage problem and because H5 pseudotypes with a polybasic cleavage site yielded trypsin-independent high RLU titers, different PCSs were inserted into H3 or H1 hemagglutinins. Unexpectedly, PCS H3 or H1 pseudotype mutants were less infectious than H5 pseudotypes (2 log_10_ RLU difference) in the absence of trypsin, in spite of the insertion of the same cleavage sequence present in H5 hemagglutinins. Besides, differences in RLU titers were observed among the several mutations tested, with a higher infectivity for the PCS4 mutants. In contrast with results obtained with IAVs [36], the results obtained in this work showed that adding a PCS in a H1 or H3 pseudotype system did not lead to efficient and sufficient trypsin-free HA processing.

Previous studies have used HAT or TMPRSS2 proteases for H1 or H3 processing either for IAVs [23] or lentiviral pseudotypes [35] [37]. In this work, we not only confirmed that these proteases independently yielded better cleavage of MCS bearing pseudotypes but that there was also a synergistic effect when both proteases were co-transfected during lentiviral production. As a result, higher titers than those described in the literature were obtained [35] [37], and the combination of the two proteases was ideal for subsequent use of pseudotypes in survival kinetics. The intracellular detection of HAT by immunofluorescence microscopy was not performed due to the lack of a HAT antibody.

It has been previously shown that IAV, though being an enveloped virus, was stable for an extended period of time in water or on surfaces and that temperature had a negative effect on viral infectivity as well as other physicochemical parameters, such as ultraviolet light (UV), salinity and pH [38] [39]. Experimental data from the present study support our previous results [10] showing that physical parameters such as temperature or salinity greatly impacted virus infectivity while the encapsidated genome remained stable over time. In addition, we previously showed that the IAV envelope was not degraded when virions were exposed to a given temperature for an extended period of time, emphasizing the role of external structures in IAV persistence outside the host [11]. Lentiviral vectors used in this study provided a simple tool to evaluate the stability in water of pseudotypes sharing the same internal proteins, envelope and genome but bearing different HAs on their surface. Lentiviral pseudotypes were affected by increased temperature and salinity, as well as their viral counterparts [10] [11]. The results showed that H1N1 NC/99 influenza pseudotypes were more susceptible to salinity at 35°C than H1N1 PAR/09 pdm pseudotypes and the same behaviour was observed with H1N1 IAVs [10]. Similarly, H3N2 pseudotypes were more influenced by high temperatures or salinities. Interestingly, H5 pseudotypes were also more stable than H3 or H1N1 NC/99 pseudotypes at 35°C in water and no persistence differences were observed when PCS H3 pseudotyped mutants were compared to their wildtype counterparts in the same experimental conditions. The main hypothesis is the possible molecular instability of the hemagglutinin protein in the presence of high salinity or temperatures, where the tertiary structure of the glycoprotein may be impacted as shown before [40]. This was confirmed by unfolding experiments or with circular dichroism, which showed that different hemagglutinin proteins behaved differently at a given temperature [40].

As a result, the loss of infectivity and the different survival trends that were observed between the different HA-bearing pseudotypes may be due to the nature of the hemagglutinin. Experiments using different HAs in recombinant IAVs are currently being performed to test this hypothesis in an actual influenza virus context. If the infectivity of recombinant viruses correlates with results obtained in this study, this could help illustrating a significant fitness factor that influences influenza virus transmission outside the host. To pinpoint the potential impact of physical or chemical factors on the infectivity of HA-bearing pseudotypes and to simplify the experimental model, these experiments were carried out in water by testing temperature and salinity. In order to better mimic environmental conditions, the combination of other physical and chemical factors, like UV light or humidity will be tested.

## Supporting Information

Figure S1 **Optimization of lentiviral pseudotype production.**
(TIFF)

Figure S2 **Persistence of H3 pseudotypes in water.**
(TIFF)

Figure S3 **Persistence of HPAI H5 pseudotypes in water.**
(TIFF)

Figure S4 **Persistence of WT H3N2 WIS/05 pseudotypes and PCS H3N2 WIS/05 mutants in water.**
(TIFF)

Table S1 **Hemagglutination assay results represented in “hemagglutination unit” (UHA) of H5N1 pseudotypes without any prior treatment or after treatment.**
(TIFF)
