# Supplementary figures and images for: Cleavage of Hemagglutinin-Bearing Lentiviral Pseudotypes and Their Use in the Study of Influenza Virus Persistence
Source: PLoS One. 2014 Aug 28;9(8):e106192. doi: 10.1371/journal.pone.0106192 (PMC4148439; doi:10.1371/journal.pone.0106192)

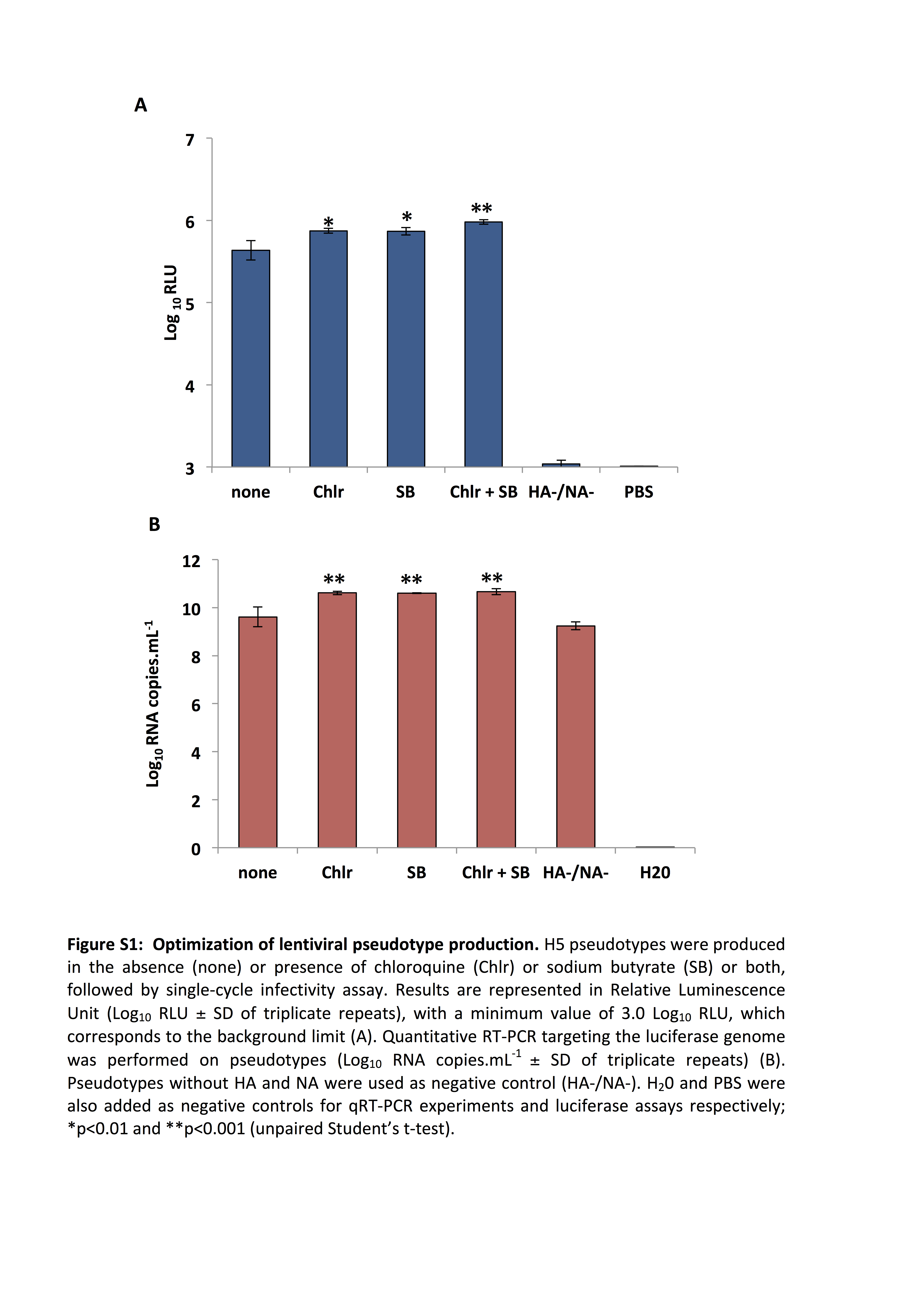

Supplement: Figure S1 — Optimization of lentiviral pseudotype production. (TIFF) [file pone.0106192.s001.tiff]

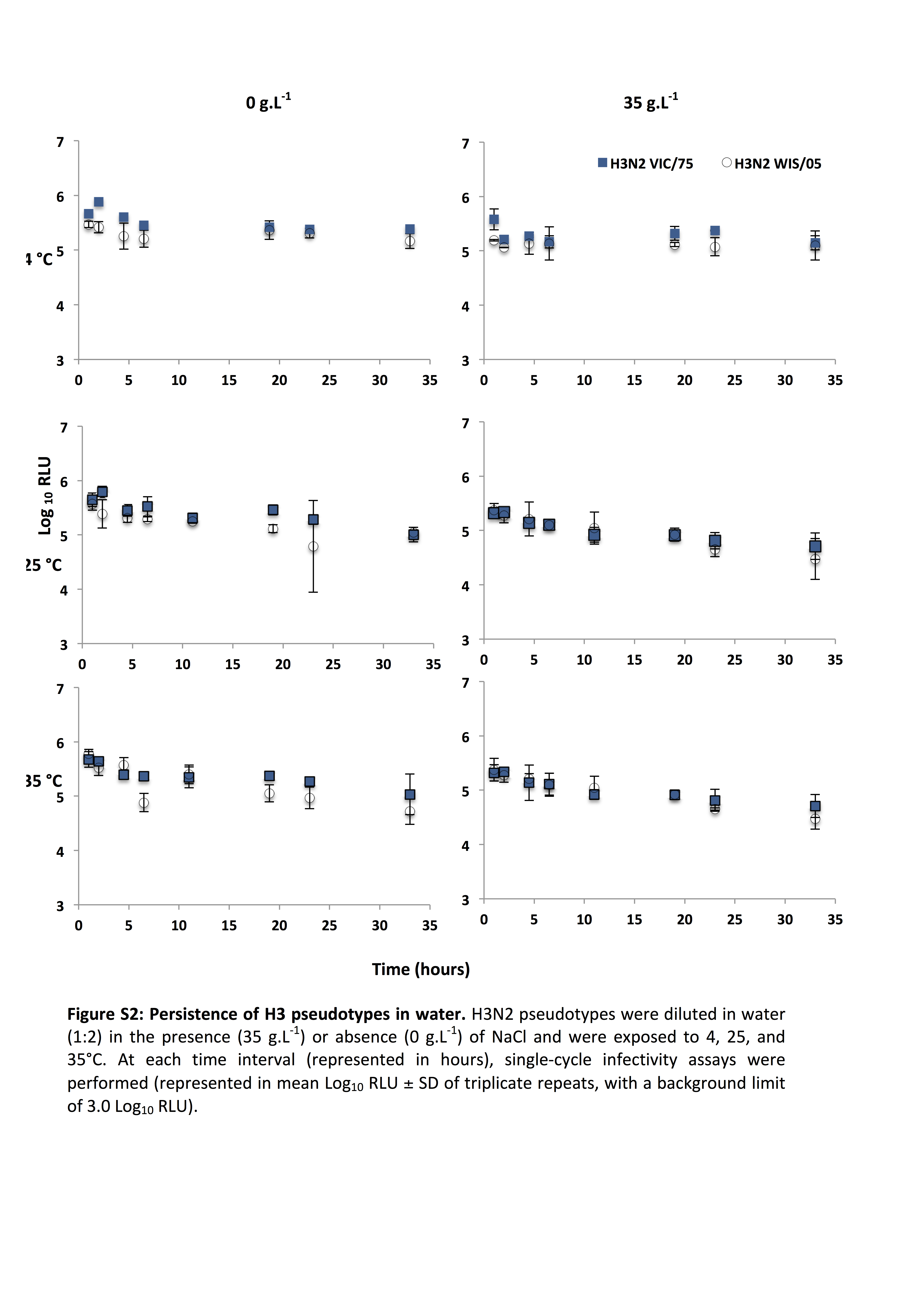

Supplement: Figure S2 — Persistence of H3 pseudotypes in water. (TIFF) [file pone.0106192.s002.tiff]

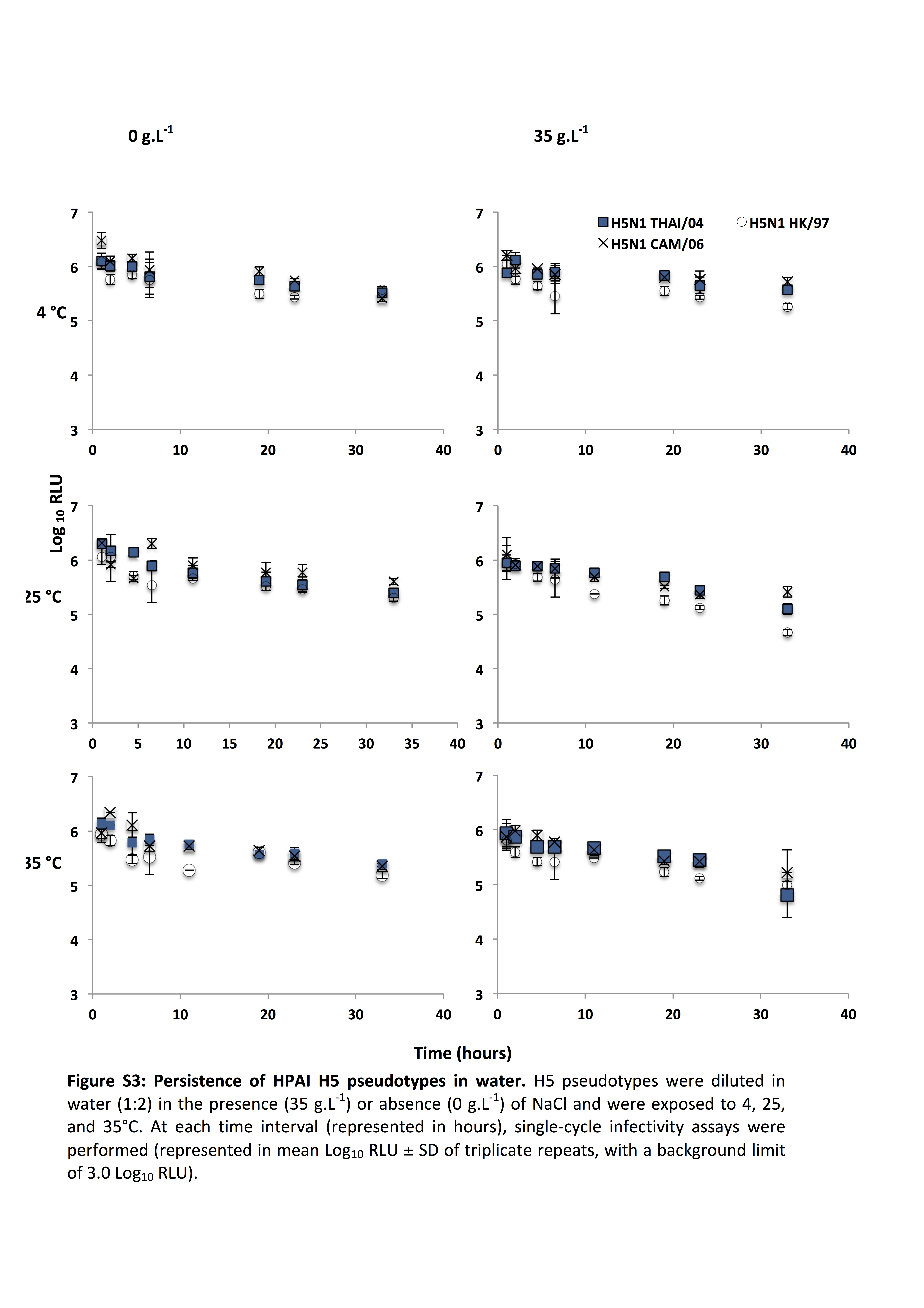

Supplement: Figure S3 — Persistence of HPAI H5 pseudotypes in water. (TIFF) [file pone.0106192.s003.tiff]

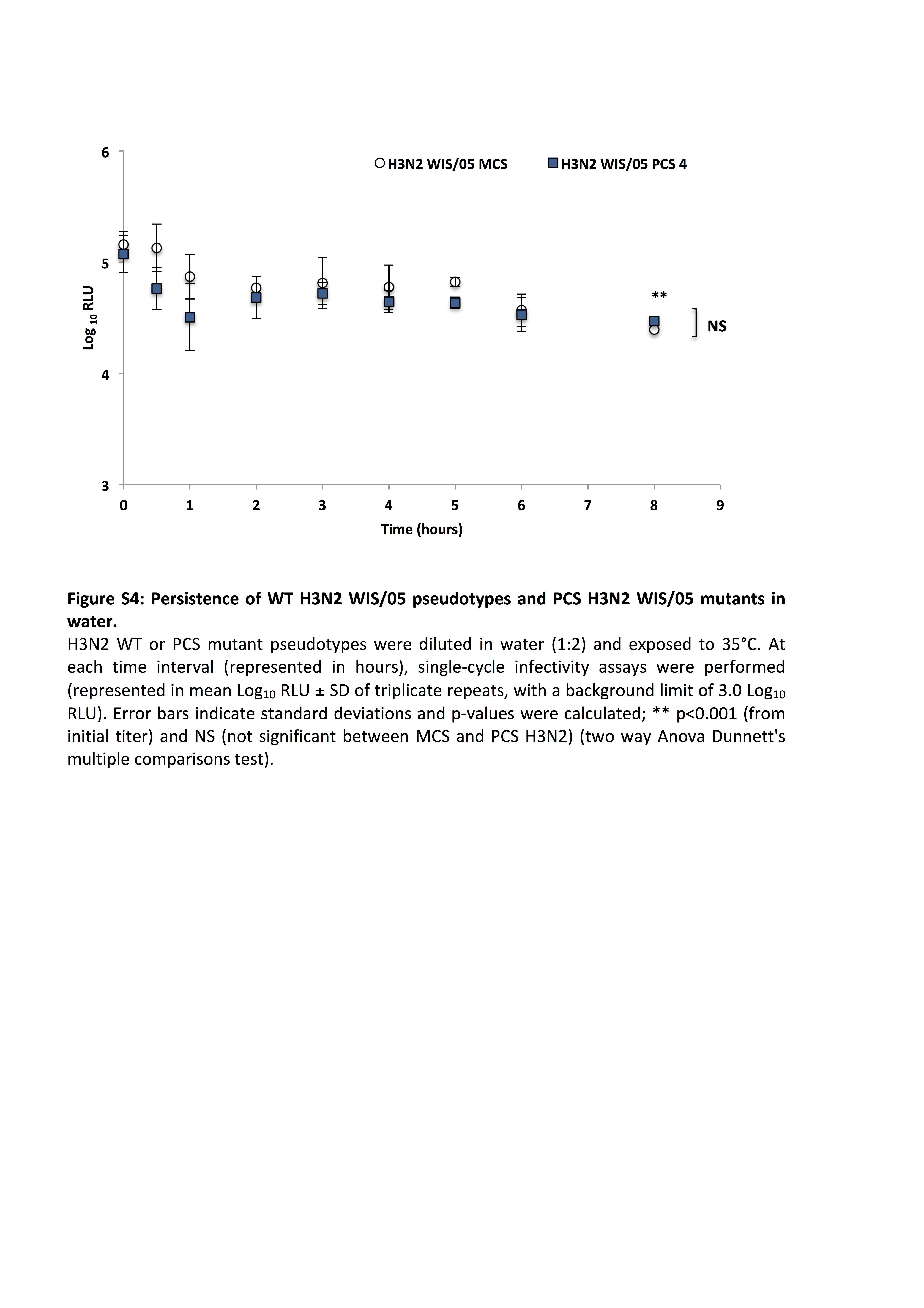

Supplement: Figure S4 — Persistence of WT H3N2 WIS/05 pseudotypes and PCS H3N2 WIS/05 mutants in water. (TIFF) [file pone.0106192.s004.tiff]

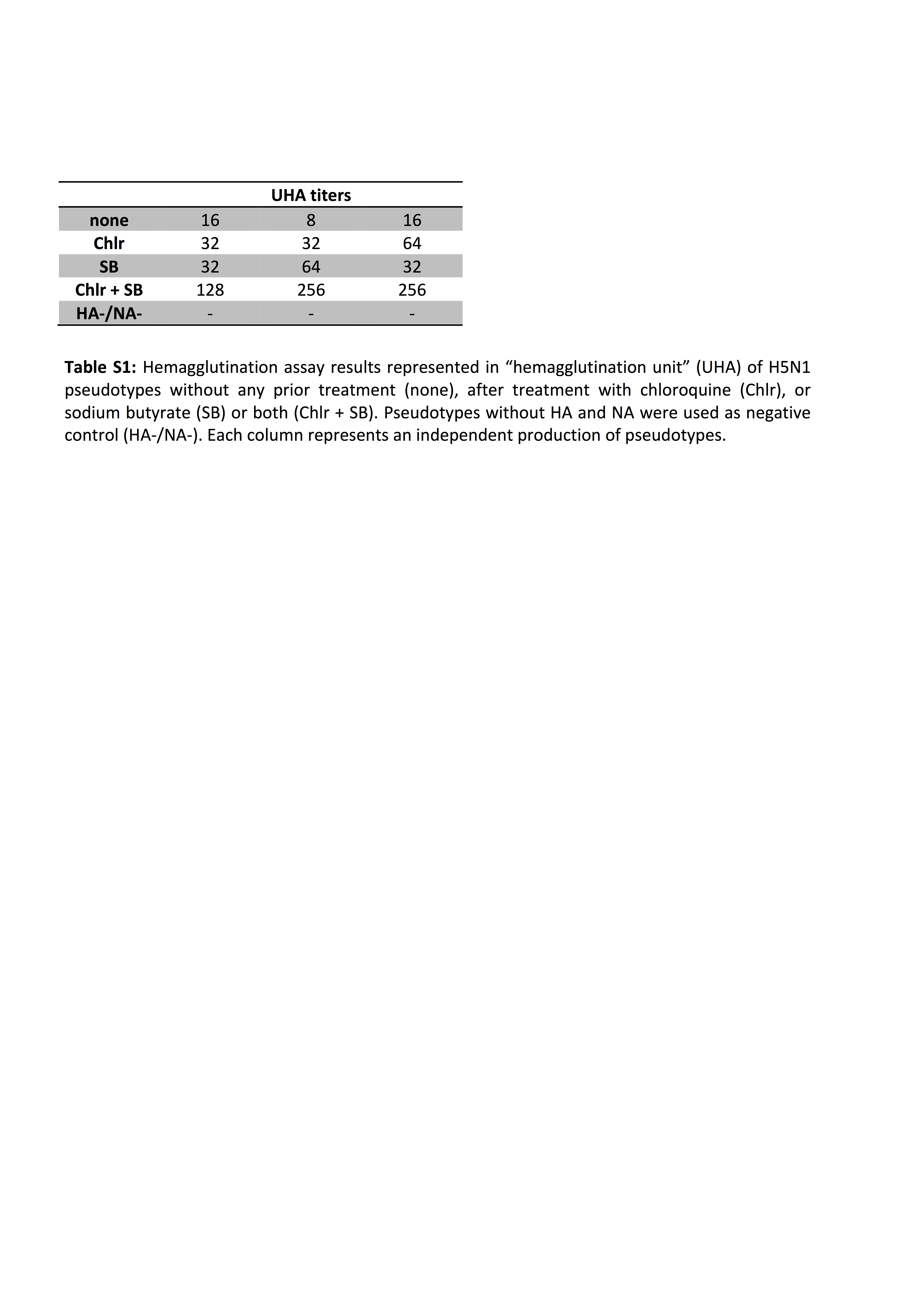

Supplement: Table S1 — Hemagglutination assay results represented in “hemagglutination unit” (UHA) of H5N1 pseudotypes without any prior treatment or after treatment. (TIFF) [file pone.0106192.s005.tiff]
